# Theoretical and Methodological Considerations for Research on Eating Disorders and Gender

**DOI:** 10.3389/fpsyg.2020.586196

**Published:** 2020-11-17

**Authors:** Marie-Luise Springmann, Jennifer Svaldi, Mechthild Kiegelmann

**Affiliations:** ^1^Department of Psychology, Karlsruhe University of Education, Karlsruhe, Germany; ^2^Department of Psychology, Clinical Psychology and Psychotherapy, University of Tübingen, Tübingen, Germany

**Keywords:** eating disorders, gender, LGBTIQ, etiology, interdisciplinary research, feminist theory, methodology

## Abstract

Gender is a relevant factor in the etiology of eating disorders (ED) as evidenced by gender-specific components of disordered eating and by the high risk of ED among transgender individuals, in addition to other factors. However, research on connections between ED and gender identity are limited. Researchers who produce explanatory models, content themselves with faulting the sociocultural ideal of slimness for women, but they fail to grasp the connection between culture, gender and the body and they fall short of integrating this perspective into existing psychological knowledge about ED. Psychological research informed by feminist theory has begun to bridge this gap, but this growing area of research needs to be further developed and should include an understanding of ED in persons with all gender identities. This article expands the discussion of gender and ED, by grounding ED in an understanding of gender itself and by discussing methodological implications of this understanding.

## Introduction

Data on the prevalence of eating disorders (ED) indicate that women are disproportionately affected, relative to men (Keski-Rahkonen and Mustelin, 2016; Udo and Grilo, 2018). It has been argued that ED in males might be underdiagnosed due to stigmatization and different symptomatology (Stanford and Lemberg, 2012; Strother et al., 2012). Body dissatisfaction in males is concerned with a well-defined muscular body ideal rather than with slimness *per se*, which suggests different patterns of eating and exercise (Murray et al., 2017). ED in persons whose gender identity and/or sexual orientation does not match binary (e.g., male and female) and heterosexual norms have received increased attention. For example, researchers found a higher risk for disordered eating among gay or bisexual men relative to heterosexual men (Gorrell and Murray, 2019). Research results for lesbian or bisexual women are less clear, but data point to a risk that is comparable to or even higher than has been found among heterosexual women (Meneguzzo et al., 2018). Transgender or non-binary persons have shown an especially high risk for intense body dissatisfaction and for ED (Jones et al., 2016; Feder et al., 2017). Thus, data show that the levels of risk for ED, as well as symptomatology, differ between genders and sexual orientations. Yet, a comprehensive explanation for the role of these factors is missing from explanatory models.

## Explanatory Models and the Question of Gender

Decades ago, publications informed by feminist perspectives drew attention to the high value society places on physical beauty when determining a women’s social status, as well as to the increasingly rigid standards of slimness and their effects on women’s self-evaluations (Boskind-Lodahl, 1976; Rodin et al., 1984; Striegel-Moore et al., 1986). In today’s multicausal model of ED etiology (Culbert et al., 2015), slimness idealization has been established as a sociocultural factor that fosters body dissatisfaction and dieting, especially in women. As MacSween (1995) has pointed out, psychological models employ this sociocultural factor as an add-on to biological, cognitive, or psychodynamic explanations that in themselves cannot account for prominent gender differences and that do not provide an integrated understanding of the role of gender.

Cognitive-behavioral models focus on the cognitive experience of eating disordered persons and frame their behaviors as logical consequences of a pattern of self-evaluation based on their ability to control eating, shape and weight (Fairburn et al., 2003). There is evidence that the transdiagnostic model of ED maintenance can be applied to men with ED (Dakanalis et al., 2014). Concerning etiological assumptions and the question of gender, it makes sense that a sociocultural ideal of slimness is a relevant factor in cognitive patterns of self-evaluation, but scholars have not further examined if and/or how this ideal actually explains the gender specificity of ED. Indeed, intraindividual explanations of cognitive models fail to answer the question, “Why it is predominantly women who feel that their personal worth is determined by their bodies and that they can access power and control by suppressing their own needs?”

Sociocultural models have provided solid empirical evidence for the relevance of sociocultural ideals of attractive bodies (body ideals), their mediation and internalization, and offer explanations for differences in symptom presentation between females and males based on different body ideals (Tylka, 2011; Stice, 2016; Klimek et al., 2018). Although the development of sociocultural models is influenced by and overlaps with research specifically addressing the question of gender (Striegel-Moore et al., 1986), the perspective of people who work with sociocultural models is often limited by their focus on beauty ideals alone and by their oversight of other ways that sociocultural context might be relevant, especially when addressing the question of gender.

Concerning biomedical models, scholars working with feminist or sociocultural models have often criticized that this perspective does not live up to the complexity of ED (Levine and Smolak, 2014). In fact, there is no compelling evidence for a gender-specific biological causality. However, there is strong evidence for the relevance of biological factors in the etiology and symptomatology of ED (Bulik et al., 2015; Culbert et al., 2015; Steinglass et al., 2019). We argue that advances in understanding of the interactions between sociocultural factors and gender specific biological developmental processes could provide promising insights (Klump et al., 2012; Murnen and Smolak, 2015; Riva et al., 2015).

Thus, although body ideals may be the most obvious connection to gender, the relevance of gender for ED could be analyzed more in depth. Extensive feminist literature on ED in social sciences postulates interrelations between social structures, gender norms and body image (Bordo, 1993; MacSween, 1995). Feminist scholars often work with social constructionist and poststructuralist accounts, whereas most psychological research is based on a rather positivist research paradigm, which often relies on a medical risk-disease model of ED. Therefore, bringing these lines of research together touches many fundamental theoretical and methodological debates. The strong focus of feminist theories on the social structures and discourses in which disordered eating occurs (and is considered as such) is in conflict with the notion of ED as an intraindividual problem that is inherent in a medical model, even if this model acknowledges social factors. Feminist authors have therefore criticized the medicalization of ED and the inherent power dynamics (MacSween, 1995; Gremillion, 2003; Walsh and Malson, 2010). This article cannot resolve these issues, but aims to point out ways in which feminist theory could further enrich current theories and research.

## Feminist Perspectives and Psychological Research

The basis of a feminist viewpoint is to understand gender as a social construct that ties certain meanings and expectations to different bodies. Gender in this perspective viewed as more of a social position than as an individual trait. Based on their bodily features, people are placed in different categories (e.g., male and female) that are associated with different social experiences. Understanding what it means to be male, female, transgender or non-binary is not possible without analyzing social structures and discourses that give meaning to these terms and influence the lived experience of a person from the very start.

Orbach’s theory (Orbach, 1986) is based on the psychodynamic approach to ED: in this perspective, ED are based on a failure to develop an autonomous self and represent a struggle to experience control. However, Orbach expands on this approach by applying a feminist framework that acknowledges gender specific social demands and developmental processes (see also Gilligan and Richards, 2018): Orbach’s theory of ED centers on an ideal of femininity that demands that women define themselves in relation to others and that that they address the needs and expectations of others. The suppression of one’s own needs or aims is therefore necessary to fulfill the female gender role. Accordingly, a position of relative social powerlessness is inscribed in the female psyche through socialization, despite the fact that women are technically, legally equal to men. Although a focus on gender roles may seem outdated, research indicates that people today still attribute communal traits to women and agentic traits to men (Ellemers, 2018). Relations between female gender role and ED symptomatology has been studied to some extend in the 1980s and 1990s. In a metaanalysis, Murnen and Smolak (1997) detected a significant, small positive effect of female gender role orientation and a small negative effect of male gender role orientation, despite very heterogeneous results of the individual studies. As has been argued elsewhere (Murnen and Smolak, 1997; Springmann, 2018), results of the individual studies were dependent on the operationalization of gender in specific questionnaires whose suitability to reflect all important aspects of such a complex construct can be contested. Here, we argue that it is less the personal acceptance of a given gender role that is important, but rather, the specific psychological traits that are fostered by socialization according to that role. Psychological research has gathered cross-sectional evidence of the importance of silencing one’s own emotions and needs in order to maintain relationships as a factor in disordered eating (Buchholz et al., 2007; Geller et al., 2010; Norwood et al., 2011), although longitudinal data are needed.

The definition of gender as a social position into which a person is placed, based on their body, informs the ways that gendered expectations and restrictions may foster a body-centered psychopathology such as ED. Feminist authors like sociologist MacSween (1995) and philosopher Bordo (1993) have written extensively about the entanglement of social constructions of bodies, selfhood, and gender. Both draw on a dualism between mind and body that is deeply rooted in Western philosophy and that values the control of mind over body. This dualism is also gendered, associating masculinity with the mind and femininity with the body. Historically, in Western culture, men are associated with reason, agency and an instrumentalized, controlled body, whereas women are depicted as more entangled in the biological sphere, passive, and irrational (MacSween, 1995). Therefore, women are more defined by their bodies and seen as objects for the active male to act on. In this perspective, it makes sense that women would consider bodily self-control as a way to achieve agency and subjectivity. Controlling their bodies could be seen as compensating for the passive and objectified qualities they are attributed due to their female bodies. The value of slimness for women is then to embody socially idealized autonomy without violating the demands of the female gender role to be desirable and submissive. Psychological work on ED and gender by Striegel-Moore et al. (1986) has already acknowledged gender differences in the way women and men relate to their bodies (appearance evaluation versus functionality). Yet, the connection between beauty ideals and the pursuit of control or autonomy remained an unanswered question. Feminist theories like those of Bordo (1993) and MacSween (1995) illustrate how gendered body ideals are the embodiment of meanings, attributed to a gender, that change with historical circumstances, rather than random ideas of beauty produced by the media.

Psychological research and theory is needed to transform ideas like these from the level of abstract cultural dynamics to concrete individual experiences that are empirically accessible and that can guide prevention and treatment. This has been done by Fredrickson and Roberts (1997), who reviewed data on social processes by which women are objectified and theorized about the psychological consequences. They proposed that girls learn to constantly view themselves through an evaluating outside perspective and to monitor their bodily appearance. This objectified self-awareness is expected to lead to feelings of anxiety and shame, to a disconnection from inner bodily signals and to a reduced capacity for other mental processes (see also Way et al., 2018). Evidence for the relevance of objectification for ED has been gathered in cross-sectional as well as a longitudinal research (Piran and Cormier, 2005; Dakanalis et al., 2015). Riva (2012); Riva et al. (2015) proposed a theoretical integration of objectifying social experiences and neurocognitive processes that result in a body perception that is ‘locked’ to a negative outside perspective.

The developmental theory of embodiment (Piran, 2016, 2017) adds fundamentally to the psychological understanding of interactions between social dynamics, gender and the body. Most psychological research draws on a biomedical understanding of the body and studies the relationship persons have with their bodies via a cognitive-perceptual concept of body image. In contrast, the phenomenological concept of embodiment refers to the lived bodily experience of engaging with the world and describes the integration of body and self (McBride, 2019). The urge to control or even fight against one’s own body, therefore, is characteristic for disembodiment. The development of embodiment in women has been found to relate to three social factors: physical freedom, mental freedom and social power (Piran, 2017). Thus, restrictions on movement and bodily expression, sexual violation, stereotyping and marginalization are theorized as destructive social processes. Namely, such social processes serve as a conduit through which gendered body ideals and sexism contribute to a disintegration of the body and the self and shape negative experiences of the body and bodily needs. This perspective adds to objectification theory by focusing less on the evaluation of bodily appearance and broadening the scope of social processes relevant for bodily experience.

## Theoretical Implications for Diverse Genders and Sexual Minorities

Risk factors for males discussed in the literature include higher than average BMI, sexual abuse and other victimization, engagement in sports that emphasize weight and body shape, or belonging to sexual or ethnic minorities (Ricciardelli and McCabe, 2004; Murray et al., 2017). Applying embodiment theory, it seems that men develop ED when they are in positions of bodily evaluation, objectification, social disempowerment and discrimination. The lower prevalence of ED in males than in females could be explained by the general assumption that female gender, as a social position, is more likely to be impacted by social dynamics of objectification and disempowerment. Psychologists may benefit from initiatives to gather more knowledge on embodiment processes in males in order to understand how the discussed risk factors might influence ED symptomatology. Moreover, cultural constructions of masculinity could be relevant to embodiment processes: In a qualitative study, Quiniones and Oster (2019) discussed striving for muscularity or weight loss as ways to deal with constructions of masculinity.

Researchers of ED in sexual and gender minorities stress the relevance of stigmatization and its psychological consequences (Bell et al., 2019). Scholars have established the fact that minority stress leads to psychological strain (Meyer, 2003), but in terms of ED, it is important to understand how minority stress influences a person’s relationship with their body (Bosley, 2011). The high risk of ED for gay men is often attributed to the high relevance of physical attractiveness in the gay community. More qualitative studies, like the one conducted by Drummond (2005), that acknowledge the subjective experience of affected persons, may help increase granularity and precision of scholars’ understanding about how bodily appearance becomes crucial for social minority men to navigate between different norms and social expectations. Gendered body ideals and the embodiment of gender roles may have different relevance to gay persons of all genders, given that normative gender roles are based on a heterosexual bisection of masculinity and femininity as complementary constructs. For example, a “butch lesbian” with a rather male gender expression may be perceived as highly attractive in her community, but may face devaluation or even hostility in normative culture. Therefore, the way she physically expresses herself might be liberated from normative beauty ideals, but arriving at the place of free expression may not have come easy for her. For others, it might be important to express their female or male gender in accordance with normative beauty ideals despite the underlying assumption that their homosexual or bisexual identity exempts them from compliance with these ideals. Alignment with gender norms might match their personal preferences and/or might enable them to avoid homophobic aggression. Concerning gay women, social factors relevant for the development of ED include experiences associated with belonging to a sexual minority group as well as experiences associated with female gender (Mason et al., 2018).

For transgender persons, the experience of incongruence between their gender identity and their bodies is associated with body discomfort that can be reduced by gender confirming medical interventions (Jones et al., 2016). Yet it might not be satisfaction with one’s bodily appearance *per se* that is relevant here, but the social meaning of being able to embody one’s core identity. Understanding the experience of being transgender or non-binary in a society with precise norms of masculinity, femininity, and the appropriate bodies for these two categories, is crucial. Otherwise, scholars risk framing the high risk for body discomfort as part of the non-normative gender identity, that is understood as an individual factor, instead of a problem grounded in the social context (Springmann, 2018). As Testa et al. (2017) demonstrated, the effect of gender confirming interventions on body satisfaction is mediated by reduced non-affirmating social experiences (e.g., being referred to with incorrect pronouns). Various studies have highlighted the general importance of social acceptance versus discrimination for ED pathology in transgender and non-binary persons (Watson et al., 2017; Bell et al., 2019). Goldhammer et al. (2019) have outlined ways to address minority stress issues in interventions.

Thus, for the same reasons that feminist authors have criticized a medical model of intraindividual psychopathology for ED in women, a lack of understanding of the social experience of sexual and gender minorities and their own perspective on their eating behaviors might foster pathologization of these persons. Although there has been increased attention to ED mental health literacy (Bullivant et al., 2020) little is known about awareness and understanding of ED in these specific populations. Further research along these lines is needed to improve prevention and health education.

## Conclusion

Understanding the role of gender for ED relies on adequate theoretical and methodological access to the construct of gender. Feminist theory has presented gender as a key construct that is not just a trait of an individual. Rather, the construct of gender is only understandable by integrating the levels of cultural structures and discourses, specific social processes that are embedded in a given cultural context, the individual experience and engagement with the sociocultural context, and the body as the basis and central medium for this engagement. The suggested transdisciplinary approach to ED in this paper allows researchers to identify specific processes or experiences related to gender that might be important for ED (e.g., objectification), but that do not necessarily affect all persons with similar gender identities in the same way. Psychological research on ED could benefit from the proposed transdisciplinary feminist approach when it is used as a starting point to better understand the relevance of social structures in the etiology and maintenance of ED. Qualitative methods could enrich the understanding of ED because they allow access to subjective experience and meaning by documenting how social pressures get translated into real life experiences. When operationalizing gender in quantitative measures, it is important to take into account: which level of the gender construct is reflected by the chosen measure, if this is actually the adequate approach for the research question, and if it contains normative biases (e.g., not reflecting gender diversity). Thus, theoretical complexity and diversity of methods are likely to advance scholars’ etiological understanding of ED in general, since they allow for important integration of social, psychological, and biological factors.

## Data Availability Statement

The original contributions presented in the study are included in the article/supplementary material, further inquiries can be directed to the corresponding author.

## Author Contributions

M-LS drafted the article. JS and MK contributed to several critical revisions. All authors approved the final version.

## Conflict of Interest

The authors declare that the research was conducted in the absence of any commercial or financial relationships that could be construed as a potential conflict of interest.
